# Limited Applicability of GW9662 to Elucidate PPAR*γ*-Mediated Fatty Acid Effects in Primary Human T-Helper Cells

**DOI:** 10.1155/2014/149628

**Published:** 2014-06-25

**Authors:** Anke Jaudszus, Stefan Lorkowski, Michael Gruen, Alexander Roth, Gerhard Jahreis

**Affiliations:** ^1^Department of Physiology and Biochemistry of Nutrition, Max Rubner-Institut, Federal Research Institute of Nutrition and Food, 76131 Karlsruhe, Germany; ^2^Department of Nutritional Biochemistry, Institute of Nutrition, Friedrich Schiller University, 07743 Jena, Germany; ^3^Department of Nutritional Physiology, Institute of Nutrition, Friedrich Schiller University, 07743 Jena, Germany; ^4^Food GmbH, 07743 Jena, Germany

## Abstract

Synthetic antagonists of the nuclear receptor PPAR*γ* such as GW9662 are widely used to elucidate receptor-mediated ligand effects. In addition and complementary to recent work, we examined whether GW9662 is suitable to serve for mechanistic investigation in T-helper cells. Human peripheral blood mononuclear cells (PBMC) were preincubated with increasing concentrations of GW9662 (0, 0.4, 2, and 10 *μ*mol/L) 30 min before adding the *c*9,*t*11-isomer of conjugated linoleic acid (*c*9,*t*11-CLA) as representative of PPAR*γ*-activating fatty acids with immunomodulatory properties. Corresponding cultures were incubated with GW9662 in the absence of the fatty acid. After 19 h, cells were mitogen stimulated for further 5 h. Subsequently, intracellular IL-2 was measured in CD3^+^CD4^+^ lymphocytes by means of flow cytometry. 100 *μ*mol/L *c*9,*t*11-CLA reduced the number of T-helper cells expressing IL-2 by 68%. GW9662 failed to abrogate this fatty acid effect, likely due to the fact that the compound exerted an own inhibitory effect on IL-2 production. Moreover, GW9662 dose-dependently induced cell death in human leukocytes. These results suggest that application of GW9662 is not conducive in this experimental setting.

## 1. Introduction

During the last decades, the scientific knowledge about the role of peroxisome proliferator-activated receptors (PPARs) in controlling metabolic and inflammatory processes has increased steadily. Among the three isoforms of the PPAR family, designated PPAR*α* (NR1C1), PPAR*β*/*δ* (NR1C2; NUC1), and PPAR*γ* (NR1C3), the latter has been specifically implicated in the regulation of immune cell function, for example, in macrophages [1] and T-helper cells [2]. In T-helper cells, the predominately expressed splice variant *γ*1 is inducible by agonist ligation [3]. Its activation by ligand binding antagonizes the proinflammatory capability of several transcription factors such as nuclear factor-*κ*B (NF-*κ*B), signal transducer and activator of transcription (STAT) [4, 5], and nuclear factor of activated T cells (NFAT) to control the expression of immunostimulatory cytokines such as IL-2 and IL-4 [6, 7].

Due to their ability to activate PPAR*γ* with micromolar affinity [8], conjugated linoleic acids (CLA), naturally occurring fatty acids in ruminant fats, aroused scientific interest as potentially anti-inflammatory agents. For instance, we have previously shown that the predominant natural isomer* c*9,*t*11-CLA reduces expression of the chemokine IL-8 in airway epithelial cells [9], inhibits IL-2 and TNF-*α* in T-helper cells [10], and prevents experimentally induced airway inflammation in mice at least in part via a PPAR*γ*-dependent mechanism [11].

GW9662 is widely used to elucidate PPAR*γ*-dependent anti-inflammatory mechanisms* in vitro* [12, 13] and* in vivo *[14–16]. This molecule covalently modifies the ligand-binding domain by arylation on the cysteine residue Cys^285^ [17] and thereby inhibits irreversibly ligand binding to and activation of PPAR*γ*.

In the present study, which is complementary to previously published work of our group [10], we examined whether GW9662 is suitable to explain PPAR*γ*-mediated effects of* c*9,*t*11-CLA in primary human T-helper cells.

## 2. Materials and Methods

### 2.1. Chemicals

Lyophilized 2-chloro-5-nitrobenzanilide (GW9662) was solubilized in sterile dimethylsulfoxide (DMSO) according to the manufacturer's instruction (Enzo, Lörrach, Germany, and Sigma, Taufkirchen, Germany) and stored in aliquots at −20°C.* c*9,*t*11-CLA (Matreya LLC, Pleasant Gap, USA) in free fatty acid form, phorbol 12-myristate 13-acetate (PMA), ionomycin, and brefeldin A (all Enzo) were likewise dissolved in DMSO, aliquoted, and stored at −20°C.

### 2.2. Purification of PBMC

Mononuclear cells were isolated from buffy coats obtained from peripheral blood of healthy volunteers who gave their written consent for blood donation. Buffy coat blood was 1 : 1 diluted with PBS (PAA, Cölbe, Germany), layered onto Lymphocyte Separation Medium (LSM) 1077 (1.077 g/mL; PAA; ratio 1 : 1), and centrifuged at 700 ×g for 20 min at 20°C. The PBMC interphase was collected, washed three times with PBS, and resuspended in RPMI 1640 medium supplemented with 10% FBS Gold (PAA).

### 2.3. Cytokine Production

PBMC (1 × 10^6^/mL) were preincubated for 30 min without or with different concentrations of GW9662 (0.4, 2, and 10 *μ*mol/L) before 100 *μ*mol/L* c*9,*t*11-CLA was added. After 19 h of incubation, cells were stimulated with PMA (2.5 ng/mL) and ionomycin (0.5 *μ*g/mL) in the presence of brefeldin A (5 *μ*g/mL) for another 5 h. Control cultures contained maximum 0.2% DMSO. Afterwards, aliquots were stained with anti-human CD3 mAb (PE-Dy647, clone MEM-57, Immunotools, Friesoythe, Germany) and anti-human CD4 mAb (FITC, clone MEM-241, Immunotools) before cells were fixed with 2% formaldehyde (Histofix, Roth, Karlsruhe, Germany). For intracellular cytokine quantification, cells were permeabilized by washing with PBS/0.1% BSA/0.1% saponin, stained with anti-human IL-2 mAb (PE, clone MQ1-17H12, eBioscience), and analyzed in reference to FMO-controls by means of flow cytometry. Nonspecific fluorescence was controlled by incubation with isotype-matched antibodies. Data were assessed and illustrated by WinMDI v.2.8 software (J. Trotter, Scripps Research Institute).

### 2.4. Cell Viability

To assess the impact of GW9662 on cell viability, PBMC (1 × 10^6^/mL) were incubated without or with 0.4, 2, and 10 *μ*mol/L of this compound for 19 h, followed by 5 h stimulation with PMA (2.5 ng/mL) and ionomycin (0.5 *μ*g/mL) in the presence of brefeldin A (5 *μ*g/mL). Control cultures contained the according volume of DMSO. Cell viability was analyzed by annexin-V (Immunotech, Marseille, France) and propidium iodide (PI; Sigma-Aldrich, Munich, Germany) exclusion double staining as previously described [10].

### 2.5. Statistics

Differences in the percentages of IL-2 positive cells were evaluated using a linear mixed model with the fixed factors “fatty acid treatment” (*c*9,*t*11-CLA and DMSO) and “PPAR*γ* antagonist treatment” (GW9662 and control) and the interaction of these two factors. The assumption of normality and homoscedasticity was justified by visual inspection of QQ-plots and predicted versus residual plots. A random intercept specific for each subject was included to control for interindividual differences. Tukey-Kramer was conducted as posthoc test and *P* values were adjusted for multiple comparisons. For evaluation of data obtained in the absence of* c*9,*t*11-CLA, the concentration of GW9662 was entered into the model as fixed factor while IL-2 positive cells, MFI, and viability were defined as dependent variables, respectively. Because the distribution of viability was skewed, a log-transform was applied. For the latter outcome, differences between concentrations 0 *μ*mol/L and 0.4 *μ*mol/L were additionally evaluated by defining posthoc contrasts between these two concentration levels. Significance of difference was set at *P* < 0.05. All calculations were carried out using SAS 9.3 (PROC MIXED).

## 3. Results

### 3.1. GW9662 Fails to Abrogate the Inhibitory Effect of* c*9,*t*11-CLA on IL-2 Expression in T-Helper Cells

**Figure 1:**
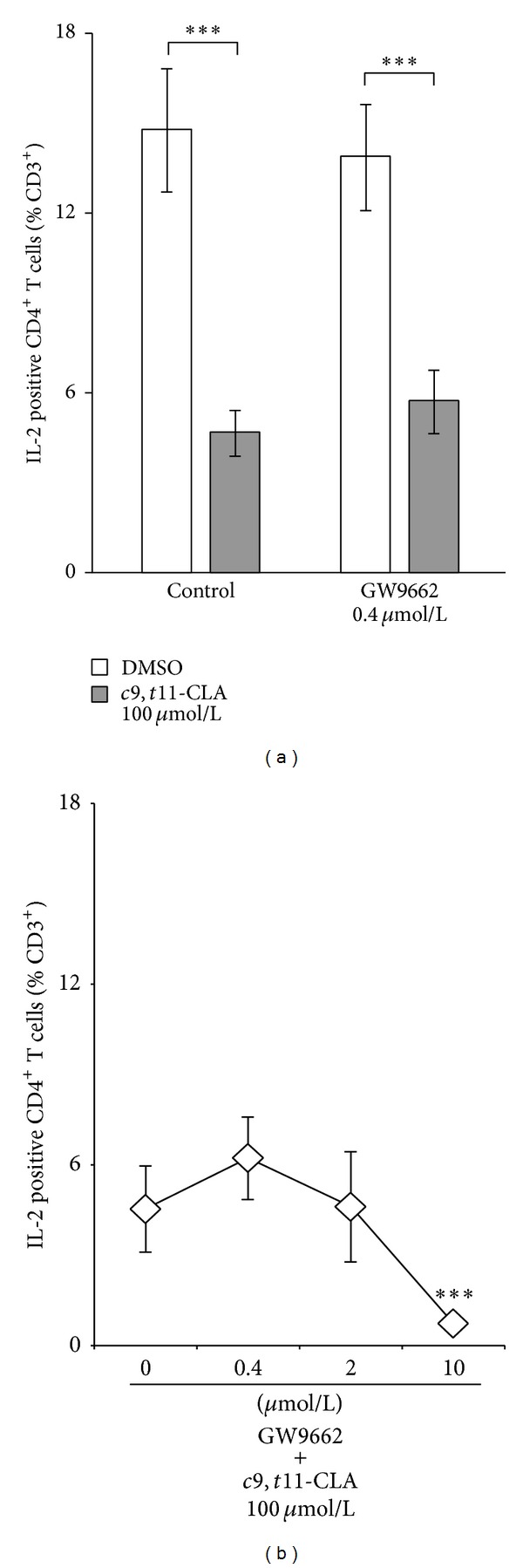
GW9662 exerts no effect up to 2 *μ*mol/L and an additive effect on IL-2 inhibition in T-helper cells at 10 *μ*mol/L. PBMC were pretreated for 30 min with GW9662 before 100 *μ*mol/L* c*9,*t*11-CLA was added. After 19 h, cells were activated for subsequent 5 h. Intracellular IL-2 was flow cytometrically analyzed in lymphocytes gated for CD3 and CD4. ***P ≤ 0.001. Data are expressed as means ± SEM of *n* = 6 (a) and *n* = 5 (b).

In stimulated control cultures, 15 ± 2% of the T cells (CD3^+^) were identified as IL-2 positive T-helper cells (CD3^+^CD4^+^; Figure 1(a)). Incubation with 100 *μ*mol/L* c*9,*t*11-CLA for 24 h significantly reduced the intracellular content of IL-2 in stimulated T-helper cells by 68% to 5 ± 1%. Preincubation with 0.4 *μ*mol/L GW9662 did not result in reexpansion of the IL-2 positive T-helper cell population. This was unexpected as preincubation with 0.4 *μ*mol/L of the PPAR*γ* antagonist T0070907, a compound with similar molecular structure to GW9662 except for one single N atom, did so in the aforementioned similar approach [10].

We further tested in a range of fivefold increases of the concentration of GW9662 whether a reversal of the fatty acid effect, in terms of blocked PPAR*γ*, was achieved. Interestingly, pretreatment with increasing concentrations of GW9662 did not lead to increased IL-2 production but even to a reduction. At 10 *μ*mol/L and in the presence of* c*9,*t*11-CLA, GW9662 caused a drop in the percentage of IL-2 positive T-helper cells even stronger than did the* c*9,*t*11-CLA treatment alone (Figure 1(b)).

### 3.2. GW9662 Dose-Dependently Downregulates IL-2 Expression in T-Helper Cells

**Figure 2:**
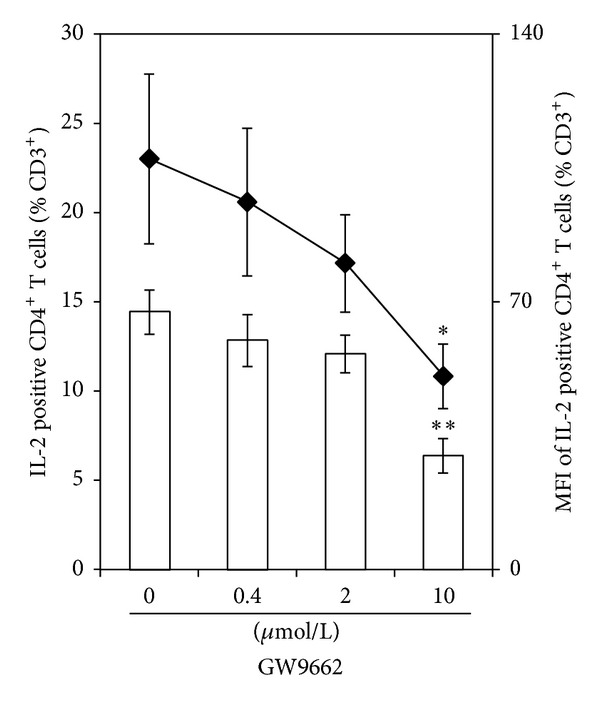
GW9662 dose-dependently downregulates IL-2 expression in T-helper cells. PBMC were incubated for a total of 24 h with increasing concentrations of GW9662. After 19 h, cells were activated for further 5 h. IL-2 expression of T-helper cells was flow cytometrically analyzed. Data are expressed as means ± SEM of *n* = 6. Right scales denote mean fluorescence intensity (MFI) depicted as aligned dots. The dose-dependent effect is statistically significant with **P < 0.01 and *P < 0.05.

We next examined whether the PPAR*γ* antagonist exerted a fatty acid independent effect itself. Indeed, with increasing concentrations of GW9662 we found a continuous reduction in the IL-2 expressing T-helper cell population. Simultaneously, mean fluorescence intensity (MFI) reflecting the cytokine levels on a per-cell basis dose-dependently decreased (Figure 2).

### 3.3. GW9662 Dose-Dependently Induces Cell Death of Human Primary Leukocytes

**Figure 3:**
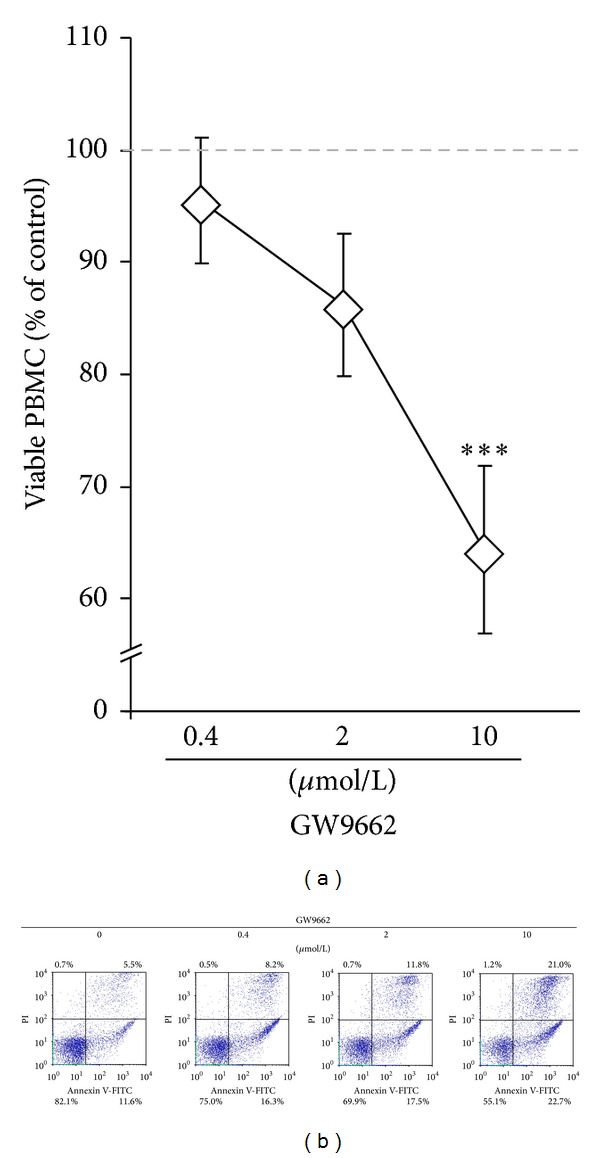
GW9662 dose-dependently causes cell death in leukocytes. PBMC were incubated for a total of 24 h with increasing concentrations of GW9662. After 19 h, stimulants were added for further 5 h. Cell viability was flow cytometrically assessed by annexin-V and propidium iodide exclusion double staining and is expressed as % of control without GW9662 (dotted line). Annexin-V positive and PI negative cells were defined as early apoptotic cells; annexin-V positive and PI positive cells were defined as late apoptotic and necrotic cells. (a) Data are expressed as means ± SEM of *n* = 4. The dose-dependent effect is statistically significant with ***P < 0.001. (b) Representative dot plots of GW9662 treated PBMC, gated for lymphocytes.

We further assessed whether putative cytotoxic effects underlie the failure of GW9662 to restore the cytokine production inhibited by* c*9,*t*11-CLA. As revealed by annexin-V and PI exclusion double staining, GW9662 dose-dependently caused cell death in PBMC (Figures 3(a) and 3(b)). After 24 h in the presence of GW9662, viability decreased by up to 35 ± 8% at 10 *μ*mol/L. However, at 0.4 *μ*mol/L GW9662 did not affect cell viability significantly (>95% of the control, *P* = 0.531).

## 4. Discussion

In line with previous work of our group [10], we demonstrated at first that* c*9,*t*11-CLA reduces the expression of the immunostimulatory cytokine IL-2 in T-helper cells. We have previously shown that* c*9,*t*11-CLA acts at least in part via a PPAR*γ*-mediated pathway, since low-dose cotreatment with the PPAR*γ* inhibitor T0070907 largely reverted this fatty acid effect [10]. Though intended to be likewise applicable, GW9662 failed to abrogate the fatty acid effect at all tested concentrations in the present approach. This outcome was unexpected, as a large body of evidence exists that indicates suitability of GW9662 to elucidate PPAR*γ*-dependent mechanisms when used at concentrations within the single- to double-digit micromolar range, including own results from* in vitro* studies in human epithelial cells [9]. However, we have indications that GW9662 acts differently from T0070907 not only in primary lymphocytes but also in other cells such as macrophages (unpublished findings). Nevertheless, in agreement with the literature, in a similar designed study like the one herein, GW9662 completely negated the modulating effects of* t*10,*c*12-CLA, a synthetic CLA isomer, on TNF-*α* expression in stimulated porcine PBMC [18]. However, corroborating our findings, Raman et al. recently reported in the Jurkat T-cell line that not only PPAR*γ* agonists but also its antagonists decreased the mitogen stimulated elevation in intracellular Ca^2+^, which could lead to IL-2 suppression via decreased transcriptional activity of NFAT [19].

In order to justify our data, we repeated the experiments with GW9662 purchased from different manufacturers (not shown). Since the results were comparable we can exclude that false-negative data have been produced. Besides PPAR*γ*, PPAR*α*, and PPAR*β*/*δ* are also expressed by PBMC [20, 21] and are bound and activated by CLA [22, 23]. However, it is not plausible that the fatty acid effects have been mediated through either of these isoforms, as Cys^285^, the modified residue in PPAR*γ*, is conserved among all three PPARs. Moreover, significantly higher concentrations of GW9662 are required for inhibition of ligand binding to PPAR*α* (factor ~10 over PPAR*γ*) and PPAR*β*/*δ* (factor ~600 over PPAR*γ*), respectively [17]. We clearly found that GW9662 dose-dependently exerts an own fatty acid independent diminishing effect on IL-2 production in primary T-helper cells. This finding is new and of significance since effects of the antagonist by its own might mask those which should be actually explained by its usage. Moreover, GW9662 is cell toxic in PBMC with increasing concentrations. GW9662 has previously been shown to cause apoptotic cell death in a concentration-dependent manner in oral squamous cells [24] and colon cells [25]. However, in these studies cancer cell lines were used and these cells underwent apoptosis also after treatment with T0070907 at concentrations higher than 10 *μ*mol/L. The cell death inducing effect of high doses of PPAR*γ* antagonists led to discuss them as potential therapeutic agents in the treatment of cancer [25, 26] but must be considered undesired in primary cells. However, as cell viability was not affected at 0.4 *μ*mol/L in our experiments, other effects than cytotoxic underlie the failure of GW9662 to serve for mechanistic exploration of the fatty acid effect that remains elusive.

In summary, and with the restriction that concentrations below 0.4 *μ*mol/L have not been tested, our data suggest that GW9662 is not valuable for determining the specific PPAR*γ*-mediated mode of fatty acid action in primary T-helper cells due to own regulatory and cytotoxic effects.

## References

[1] Majdalawieh A, Ro HS (2010). PPARgamma1 and LXRalpha face a new regulator of macrophage cholesterol homeostasis and inflammatory responsiveness, AEBP1. *Nuclear Receptor Signaling*.

[2] Clark RB, Bishop-Bailey D, Estrada-Hernandez T, Hla T, Puddington L, Padula SJ (2000). The nuclear receptor PPAR*γ* and immunoregulation: PPAR*γ* mediates inhibition of helper T cell responses. *Journal of Immunology*.

[3] Norazmi MN, Mohamed R, Nurul AA, Yaacob NS (2012). The modulation of PPAR*γ*1 and PPAR*γ*2 mRNA expression by ciglitazone in CD3/CD28-activated naïve and memory CD4+ T cells. *Clinical and Developmental Immunology*.

[4] Delerive P, Fruchart JC, Staels B (2001). Peroxisome proliferator-activated receptors in inflammation control. *Journal of Endocrinology*.

[5] Wang P, Anderson PO, Chen S, Paulsson KM, Sjögren H, Li S (2001). Inhibition of the transcription factors AP-1 and NF-*κ*B in CD4 T cells by peroxisome proliferator-activated receptor *γ* ligands. *International Immunopharmacology*.

[6] Yang XY, Wang LH, Chen T (2000). Activation of human T lymphocytes is inhibited by peroxisome proliferator-activated receptor *γ* (PPAR*γ*) agonists. PPAR*γ* co-association with transcription factor NFAT. *The Journal of Biological Chemistry*.

[7] Chung SW, Kang BY, Kim TS (2003). Inhibition of interleukin-4 production in CD4^+^ T cells by peroxisome proliferator-activated receptor-*γ* (PPAR-*γ*) ligands: involvement of physical association of between PPAR-*γ* and the nuclear factor of activated T cells transcription factor. *Molecular Pharmacology*.

[8] Belury MA, Moya-Camarena SY, Lu M, Shi L, Leesnitzer LM, Blanchard SG (2002). Conjugated linoleic acid is an activator and ligand for peroxisome proliferator-activated receptor-gamma (PPAR*γ*). *Nutrition Research*.

[9] Jaudszus A, Foerster M, Kroegel C, Wolf I, Jahreis G (2005). *Cis*-9,*trans*-11-CLA exerts anti-inflammatory effects in human bronchial epithelial cells and eosinophils: comparison to *trans*-10,*cis*-12-CLA and to linoleic acid. *Biochimica et Biophysica Acta—Molecular and Cell Biology of Lipids*.

[10] Jaudszus A, Jahreis G, Schlörmann W (2012). Vaccenic acid-mediated reduction in cytokine production is independent of *c*9,*t*11-CLA in human peripheral blood mononuclear cells. *Biochimica et Biophysica Acta—Molecular and Cell Biology of Lipids*.

[11] Jaudszus A, Krokowski M, Möckel P (2008). *Cis*-9, *trans*-11-conjugated linoleic acid inhibits allergic sensitization and airway inflammation via a PPAR*γ*-related mechanism in mice. *Journal of Nutrition*.

[12] An J, Nakajima T, Kuba K, Kimura A (2010). Losartan inhibits LPS-induced inflammatory signaling through a PPAR*γ*-dependent mechanism in human THP-1 macrophages. *Hypertension Research*.

[13] Marion-Letellier R, Butler M, Déchelotte P, Playford RJ, Ghosh S (2008). Comparison of cytokine modulation by natural peroxisome proliferator-activated receptor *γ* ligands with synthetic ligands in intestinal-like Caco-2 cells and human dendritic cells—potential for dietary modulation of peroxisome proliferator-activated receptor *γ* in intestinal inflammation. *The American Journal of Clinical Nutrition*.

[14] Honda K, Marquillies P, Capron M, Dombrowicz D (2004). Peroxisome proliferator-activated receptor *γ* is expressed in airways and inhibits features of airway remodeling in a mouse asthma model. *Journal of Allergy and Clinical Immunology*.

[15] Penas F, Mirkin GA, Hovsepian E (2013). PPAR*γ* ligand treatment inhibits cardiac inflammatory mediators induced by infection with different lethality strains of *Trypanosoma cruzi*. *Biochimica et Biophysica Acta—Molecular Basis of Disease*.

[16] Woerly G, Honda K, Loyens M (2003). Peroxisome proliferator-activated receptors *α* and *γ* down-regulate allergic inflammation and eosinophil activation. *Journal of Experimental Medicine*.

[17] Leesnitzer LM, Parks DJ, Bledsoe RK (2002). Functional consequences of cysteine modification in the ligand binding sites of peroxisome proliferator activated receptors by GW9662. *Biochemistry*.

[18] Kim DI, Kim KH, Kang JH (2010). Trans-10, cis-12-conjugated linoleic acid modulates NF-?B activation and TNF-a production in porcine peripheral blood mononuclear cells via a, pp. AR-Adependent pathway. *British Journal of Nutrition*.

[19] Raman P, Kaplan BLF, Kaminski NE (2012). 15-Deoxy-Δ12,14-prostaglandin J2-glycerol, a putative metabolite of 2-arachidonyl glycerol and a peroxisome proliferator-activated receptor *γ* ligand, modulates nuclear factor of activated T cells. *Journal of Pharmacology and Experimental Therapeutics*.

[20] Bouwens M, Afman LA, Müller M (2008). Activation of peroxisome proliferator-activated receptor alpha in human peripheral blood mononuclear cells reveals an individual gene expression profile response. *BMC Genomics*.

[21] Al Yacoub N, Romanowska M, Krauss S, Schweiger S, Foerster J (2008). PPAR*δ* is a type 1 IFN target gene and inhibits apoptosis in T cells. *Journal of Investigative Dermatology*.

[22] Moya-Camarena SY, Vanden Heuvel JP, Blanchard SG, Leesnitzer LA, Belury MA (1999). Conjugated linoleic acid is a potent naturally occurring ligand and activator of PPAR*α*. *Journal of Lipid Research*.

[23] Lampen A, Leifheit M, Voss J, Nau H (2005). Molecular and cellular effects of *cis*-9, *trans*-11-conjugated linoleic acid in enterocytes: effects on proliferation, differentiation, and gene expression. *Biochimica et Biophysica Acta—Molecular and Cell Biology of Lipids*.

[24] Masuda T, Wada K, Nakajima A (2005). Critical role of peroxisome proliferator-activated receptor *γ* on anoikis and invasion of squamous cell carcinoma. *Clinical Cancer Research*.

[25] Schaefer KL, Takahashi H, Morales VM (2007). PPAR*γ* inhibitors reduce tubulin protein levels by a PPAR*γ*, PPAR*δ* and proteasome-independent mechanism, resulting in cell cycle arrest, apoptosis and reduced metastasis of colorectal carcinoma cells. *International Journal of Cancer*.

[26] Seargent JM, Yates EA, Gill JH (2004). GW9662, a potent antagonist of PPAR*γ*, inhibits growth of breast tumour cells and promotes the anticancer effects of the PPAR*γ* agonist rosiglitazone, independently of PPAR*γ* activation. *The British Journal of Pharmacology*.

